# Geographical Distribution of Iron Redox Cycling Bacterial Community in Peatlands: Distinct Assemble Mechanism Across Environmental Gradient

**DOI:** 10.3389/fmicb.2021.674411

**Published:** 2021-05-25

**Authors:** Liang Yang, Ming Jiang, Yuanchun Zou, Lei Qin, Yingyi Chen

**Affiliations:** ^1^Northeast Institute of Geography and Agroecology, Chinese Academy of Sciences, Changchun, China; ^2^University of Chinese Academy of Sciences, Beijing, China; ^3^Jilin Provincial Joint Key Laboratory of Changbai Mountain Wetland and Ecology, Changchun, China

**Keywords:** iron redox cycling bacteria, biogeographic distribution, community assembly, peatlands, Northeast China

## Abstract

Microbial-mediated iron (Fe) oxidation and reduction greatly contribute to the biogeochemistry and mineralogy of ecosystems. However, knowledge regarding the composition and distribution patterns of iron redox cycling bacteria in peatlands remains limited. Here, using high-throughput sequencing, we compared biogeographic patterns and assemblies of the iron redox cycling bacterial community between soil and water samples obtained from different types of peatland across four regions in Northeast China. A total of 48 phylotypes were identified as potential iron redox bacteria, which had greater than 97% similarity with Fe(II)-oxidizing bacteria (FeOB) and Fe(III)-reducing bacteria (FeRB). Among them, *Rhodoferax*, *Clostridium*, *Geothrix*, *Sideroxydans*, *Geobacter*, *Desulfovibrio*, and *Leptothrix* could be used as bioindicators in peatlands for characterizing different hydrological conditions and nutrient demands. Across all samples, bacterial communities associated with iron redox cycling were mainly affected by pH, dissolved organic carbon (DOC), and Fe^2+^. Distance–decay relationship (DDR) analysis indicated that iron redox cycling bacterial communities in soil, but not in water, were highly correlated with geographic distance. Additionally, null model analysis revealed that stochastic processes substituted deterministic processes from minerotrophic fens to ombrotrophic bogs in soils, whereas deterministic processes were dominant in water. Overall, these observations suggest that bacteria involved in iron redox cycling are widespread in diverse habitats and exhibit distinct patterns of distribution and community assembly mechanisms between soil and water in peatlands.

## Introduction

The redox reactions involving iron are vital for the biogeochemical cycling of iron in terrestrial and aquatic ecosystems and are interconnected with the cycling of other elements, including carbon, nitrogen, phosphorus, sulfur, and manganese, through numerous processes in the biogeochemical iron cycle (Jickells et al., 2005; Raiswell and Canfield, 2012; Riedel et al., 2013). Owing to the redox sensitivity of iron, iron cycling occurs largely through reduction and oxidation processes. In the early 20^*th*^ century, iron redox cycling was assumed to be purely abiotic. However, the contribution of abiotic processes alone could not explain the observed spatiotemporal distribution of Fe redox cycling species in some environments, such as in the soil, plant rhizospheres, or even rust-colored flocculent mats (Melton et al., 2014). A better understanding of the biogeochemistry of ecosystems and the development of the microbiological research methods has led to the recognition that iron-metabolizing microorganisms are extremely important for biogeochemical processes globally (Emerson and Floyd, 2005; Weber et al., 2006).

Peatlands store approximately 30% of soil organic carbon and 10% of all freshwater, despite covering only 3% of the Earth’s landmass (Gorham, 1991; Yu et al., 2010). Meanwhile, different types of peatlands exhibit different redox conditions and nutrition statuses, suggesting that they are replete in carbon, nitrogen, and/or sulfur sources that are readily available for microbial metabolic and redox processes (Kügler et al., 2019). Studies have shown that Fe(II)/Fe(III) as electron donors/acceptors are widely present in peatlands (Todorova and Costello, 2006; Reiche et al., 2008). Owing to the recurrent fluctuations of the redox potential in the upper layer of peatlands, iron redox cycling microorganisms can utilize a variety of terminal electron donors (such as ammonium, humus, and hydrogen) and electron acceptors (such as nitrate and metal cations) in peatland biogeochemical cycling (Küsel et al., 2008; Lüdecke et al., 2010). Therefore, we hypothesized that microorganisms involved in iron redox cycling would be widely distributed in peatlands and that the biogeographic patterns and assembly processes would differ between peatland soil and water samples.

Fe(II)-oxidizing bacteria (FeOB) and Fe(III)-reducing bacteria (FeRB) play important roles in microbial groups capable of iron redox functions. Numerous studies have documented that these iron redox cycling bacteria are widespread in the environment, including in marine, forest, and wetland ecosystems (Haaijer et al., 2008; Dubinsky et al., 2010; Emerson et al., 2010). Simultaneously, they have important environmental implications; for example, FeOB/FeRB can be harnessed for bioremediation purposes. For instance, Fe(III)-reducing bacteria (e.g., *Geobacter*, *Shewanella*, and *Anaeromyxobacter*) or Fe(II)-oxidizing bacteria (e.g., *Gallionella*, *Leptothrix*, and *Sideroxydans*) remove contaminants by altering the valence state of iron coupled with the oxidation or reduction of organic and inorganic material, such as hydrocarbons, pesticides, explosives, and heavy metals (Herath et al., 2010; Glodowska et al., 2020; Lu et al., 2020). Also, Fe(II)-oxidizing bacteria oxidize Fe(II) to Fe(III) (oxyhydr)oxides, which detoxify the environment by absorbing or co-precipitating heavy metals (Huang et al., 2012; Smith et al., 2017; Xu et al., 2017). In addition, Kappler et al. (2021) recently reported that microbial Fe(III) reduction coupled to methane and ammonium oxidation exists in various ecosystems, and provided new insights for the comprehensive assessment of greenhouse gas (GHG). Although there is evidence to support their potential roles in biogeochemical processes (Weber et al., 2006; Melton et al., 2014), how the biogeographical distribution of FeOB/FeRB is shaped by ecological factors at regional scales remains unknown.

To date, the lack of specific primers that could encompass all iron redox cycling microorganisms has limited investigation into their biogeographical distribution (Konstantinidis and Tiedje, 2007; Parro et al., 2007). Küsel et al. (2008) and Lüdecke et al. (2010) addressed the composition of FeOB and FeRB in peatlands using a combination of molecular technology and enrichment culture. However, these studies did not elucidate the biogeographical distribution of FeOB/FeRB in peatlands, because changes in substrate type and concentration fundamentally modify the niche of these microorganisms (Yi et al., 2013; Pierra et al., 2015). Owing to its high resolution and cost-effectiveness, high-throughput sequencing (HTS) has become the method of choice for identifying microbial phylotypes (Rousk et al., 2010; Yuan et al., 2016). To open new insights into the biogeographic distribution of iron-cycling bacteria, HTS data were compared against the National Center for Biotechnology Information (NCBI) 16S rRNA database using the BLAST algorithm. Here we studied what was the geographical distribution of the iron redox cycling bacterial community and how environmental gradients affected their assemble mechanism across environmental gradients in three different types of peatland across Northeast China. Our objectives were to (i) investigate the biogeographic distribution of bacteria involved in iron redox cycling in soil and water across peatlands and (ii) identify the assembly mechanism and the driving factors of abundance and community composition of iron redox cycling bacteria in peatlands.

## Materials and Methods

### Study Sites and Sample Collection

The study sites were located in Helongjiang and Jilin provinces in Northeast China. These study sites have four regions, Sanjiang Plain (SJ), Lesser Khingan Mountain (LK), Greater Khingan Mountain (GK), and Changbai Mountain (CB), comprising three peatland types: minerotrophic fens (fen), ombrotrophic bogs (bog), and mixed peatlands (fen–bog complex). Sanjiang Plain is a temperate continental monsoon climate with seasonally frozen soil; the mean annual temperature is 2.5°C. Changbai Mountain is a temperate continental monsoon climate with seasonally frozen soil; the mean annual temperature is 3.3°C. Lesser Khingan Mountain is a temperate continental monsoon climate with island-shaped permafrost; the mean annual temperature is −1.0°C. Greater Khingan Mountain is a cold temperate continental monsoon climate with continuous permafrost; the mean annual temperature is −4.0°C (Man et al., 2010; Zhu et al., 2015; Han et al., 2018; Jiang et al., 2018). The peatlands of all sites are natural due to local protection or less anthropogenic disturbances. The soils in all sites are classified as peat soil with high content of organic matter. Detailed site characteristics are shown in Supplementary Table 1. Sampling was carried out in July 2019. Surface soil samples (30 cm) were obtained using a peat sampler (BJ7R-0409, Netherlands), and visible plant residues were removed. Water samples were extracted with a syringe and then filtered. For each site, three replicate plots were established and randomly arranged. At each plot, both the soil and water samples were collected at five sampling points and mixed to obtain one composite sample. All samples were sealed on ice and transported to the laboratory, where they were divided into three subsamples: one subsample was used for physicochemical analyses, one was transferred into 0.5 M HCl for Fe(II) determination, and one was stored at −80°C for subsequent molecular analysis.

### Measurements of Soil Physicochemical Properties

The pH was measured with a pH meter (PHS-3C, Shanghai, China) from a soil: distilled water (1:5, *w/v*) solution. Organic matter (OM) was determined by potassium dichromate oxidation-outer heating (Lu, 1999). Dissolved organic carbon (DOC) concentrations were measured in filtrates (0.4-μm filters) with a TOC-V CPN (Shimadzu, Tokyo, Japan) analyzer using high-temperature (680°C) combustion. Total nitrogen (TN) was measured with an elemental analyzer (VarioEL III, Germany). Ammonia (NH_4_^+^) and nitrate (NO_3_^–^) were extracted with 2 M KCl. Total phosphorus (TP) was assayed by HF-HClO_4_ digestion, and phosphate (PO_4_^3–^) was extracted by sodium bicarbonate extraction. NH_4_^+^, NO_3_^–^, TP, and PO_4_^3–^ were measured using a continuous-flow analyzer (Skalar Analytical BV, Breda, Netherlands). Total sulfur (TS), total manganese (TMn), and total iron (TFe) were assayed by HF-HClO_4_ digestion and 10% HNO_3_ extraction. Soil sulfate (SO_4_^2–^) was assayed from a soil: distilled water (1:5, *w/v*) solution. TS, SO_4_^2–^, TMn, and TFe concentrations were measured using the ICPS-7500 sequential plasma spectrometer (Shimadzu, Tokyo, Japan). Ferrous ion (Fe^2+^) and ferric ion (Fe^3+^) contents were analyzed using the ferrozine–ultraviolet absorbance method (Stookey, 1970). The oxidation–reduction potential (ORP) of water samples was assessed in the field using an ORP meter (PHS-JPB-607A, Shanghai, China).

### DNA Extraction, PCR Amplification, and Illumina Sequencing

Bacterial DNA was isolated from samples using the Power Soil DNA Isolation Kit (MoBio Laboratories, Inc., Carlsbad, CA, United States) according to the manufacturer’s protocol. DNA quality and quantity were assessed by the optical density at wavelength 260/230-nm and 260/280-nm ratios, respectively. The 16S rRNA V3–V4 region was amplified for each sample using the 338F (5′-ACTCCTACGGGAGGCAGCA-3′) and 806R (5′-GGACTACHVGGGTWTCTAAT-3′) primer pair. The primers were modified to contain a unique 8-nucleotide barcode at the 5′ end (Mori et al., 2014). PCR amplification and quantification details have been previously described (Yang et al., 2019a). Briefly, thermal cycling conditions of the first PCR step were as follows: an initial denaturation at 95°C for 5 min, followed by 15 cycles at 95°C for 1 min, 50°C for 1 min, and 72°C for 1 min, with a final extension at 72°C for 7 min. The thermal cycling conditions of second PCR step were as follows: an initial denaturation at 98°C for 30 s, followed by 10 cycles at 98°C for 10 s, 65°C for 30 s min, and 72°C for 30 s, with a final extension at 72°C for 5 min. Then, the amplified products were purified and recovered using the 1.0% agarose gel electrophoresis method. HTS analysis of bacterial rRNA genes was performed on purified samples using the Illumina HiSeq 2500 platform (2 × 250 paired ends). All raw sequences have been deposited into the NCBI Sequence Read Archive with the accession number SRP270023.

### Processing of the Sequencing Data

Raw Illumina fastq files were demultiplexed, quality filtered, and analyzed using QIIME 2. Sequencing reads were merged using FLASH (version 1.2.7^1^) based on overlapping regions within paired-end reads (Magoc and Salzberg, 2011). The trimmed sequences were compared to the primers, and the tags with more than six mismatches were discarded using the FASTX-Toolkit. Tags with an average quality score of <20 in a 50-bp sliding window were truncated using Trimmomatic (version 0.33), and tags shorter than 350 bp were removed. Chimeras were detected and removed using UCHIME (version 4.2). High-quality sequences were clustered using USEARCH (version 10.0), and the tags were clustered into operational taxonomic units (OTUs) at a 97% similarity threshold. Taxonomy was assigned to all OTUs by searching against the SILVA databases (release 128^2^).

Iron redox cycling bacteria were initially identified at the genus level *via* the Functional Annotation of Prokaryotic Taxa database (Louca et al., 2016). Wang et al. (2009) used the BLAST 16S rRNA database to identify novel 16S rRNA primers targeting Gallionella-related bacteria, and thus all OTU sequences of iron redox cycling bacteria at the genus level in our research were compared with existing sequences using the online BLAST 16S rRNA database^3^. Sequences showing >97% similarity with FeOB and FeRB were retained and defined as potential iron redox cycling bacteria (Weber et al., 2006; Emerson et al., 2010; Melton et al., 2014). Additionally, these bacteria of FeOB and FeRB were selected for phylogenetic tree reconstruction to clarify their taxonomic status in MEGA-X using the neighbor-joining algorithm (Kumar et al., 2018).

### Statistical and Bioinformatic Analysis

Alpha diversity indices relating to community diversity (Shannon and Simpson) and sequencing depth (Good’s coverage) were calculated by Mothur (version 1.30^4^). Alpha diversity indices were tested for differences among three peatland types and four regions by one-way analysis of variance (ANOVA) using SPSS 17.0 for Windows (IBM SPSS Inc., United States). For beta diversity, non-metric multidimensional scaling (NMDS), coupled with analysis of similarity (ANOSIM), was used to visualize the differences in composition among peatland types (fen, bog, and fen–bog complex) and regions (SJ, CB, LK, and GK), and significance tests of differences were performed using the Bray–Curtis dissimilarity. The independent influences of regions, soil physicochemical properties, and iron redox cycling bacterial community composition were assessed by Mantel’s test. To identify the main physicochemical properties that were significantly correlated with bacterial communities involved in iron redox cycling, redundancy analysis (RDA) combined with forward selection was applied using the forward.sel function in the R package. The proportion of variation in the iron redox cycling bacterial communities that could be explained by spatial variables (geographical distances) and environmental variables was calculated by variation partitioning analysis (VPA). The NMDS, ANOSIM, Mantel’s test, RDA, and VPA were conducted in the R platform with the vegan package (R Development Core Team, 2006).

Distance–decay describes how the similarity in species composition between two communities varies with the geographic distance that separates them (Morlon et al., 2008). To visualize distance–decay relationships (DDRs) of iron redox cycling bacterial community similarity and environmental dissimilarity, iron redox cycling bacterial community similarity was calculated *via* the Bray–Curtis dissimilarity and the environmental heterogeneity of each sample was calculated based on the Euclidean distance. Regarding environmental heterogeneity, except for soil pH, all other environmental variables were first standardized at the same scale and checked using the Shapiro–Wilk test and then log (*x* + 1)-transformed to improve homoscedasticity and normality (Mo et al., 2018). The microbial community similarity, environmental dissimilarity, and geographic distance matrixes were linearized using the vegan package and the DDRs were linearized in the R platform.

To investigate the community assembly mechanism (deterministic process vs. stochastic process), a null model analysis was conducted using abundance-based similarity metrics. A null model is a pattern-generating model that is based on randomization of ecological data or random sampling from a known or imagined distribution (Gotelli and Graves, 1996). Permutational multivariate analysis of variance (PERMANOVA), a non-parametric permutation test, was used to test the significance of the differences between the observed similarity matrices and the null model expectation of the iron redox cycling bacterial communities. If community assembly is primarily driven by deterministic processes, the actual bacterial communities will be significantly different from the corresponding null expectations. In contrast, if community assembly is primarily driven by stochastic processes, the actual similarity observed will be statistically indistinguishable from that of the random null expectation (Zhang et al., 2019). In addition, the standardized effect size (SES) was calculated as the differences in beta diversity between the real communities and the mean value of null communities divided by the standardized deviation of the beta diversity in the null communities. The relative importance of stochastic processes increases when SES is closer to zero (Ren et al., 2017). Null model analysis and PERMANOVA were performed using Vegan and Parallel packages in the R platform.

To identify indicative taxa, the linear discriminant analysis (LDA) effect size (LEfSe) method was used based on a normalized relative abundance matrix. The LEfSe method uses the Kruskal–Wallis test to identify the features that differed significantly among peatland types. An LDA threshold score of 4.0 and a significant *P* of 0.05 were used to detect indicator species among treatments. Significant taxa were used to generate taxonomic cladograms illustrating differences between sample genera using the website http://huttenhower.sph.harvard.edu/galaxy (Segata et al., 2011).

## Results

### Physicochemical Properties of Surface Soil and Water

The physicochemical properties of soil and water samples differed from each other (Tables 1, 2). The pH showed wide variation, ranging from 4.11 to 5.59 in soil and from 5.01 to 7.12 in water. Fe^2+^ was significantly and negatively correlated with pH (*r* = −0.451, *p* < 0.01) and NH_4_^+^ (*r* = −0.302, *p* < 0.05) and significantly and positively correlated with DOC (*r* = 0.741, *p* < 0.01) in water. In contrast, Fe^3+^ showed a significant positive correlation with pH (*r* = 0.302, *p* < 0.05) and NH_4_^+^ (*r* = 0.352, *p* < 0.05) and a significant negative correlation with DOC (*r* = −0.390, *p* < 0.01) in soil.

**TABLE 1:** Physicochemical properties of soil samples in peatlands (*n* = 3).

**Site**	**Latitude and longitude**	**pH**	**TN (g kg^–1^)**	**NH_4_^+^ (mg kg^–1^)**	**NO_3_^–^ (mg kg^–1^)**	**TP (g kg^–1^)**	**PO_4_^3–^ (mg kg^–1^)**	**OM (%)**	**DOC (g kg^–1^)**	**TS (g kg^–1^)**	**SO_4_^2–^ (mg kg^–1^)**	**Fe^2+^ (g kg^–1^)**	**Fe^3+^ (g kg^–1^)**	**TFe (g kg^–1^)**	**TMn (mg kg^–1^)**
QL	47°46′N, 132°54′E	5.56	15.38	27.83	29.15	1.95	23.13	34.27	2.58	1.40	234.67	10.65	0.32	21.48	229.19
HH2	47°47′N, 133°38′E	5.29	28.79	28.35	4.06	1.07	4.79	72.32	2.41	1.41	71.23	3.43	0.14	5.96	331.72
BL	47°32′N, 133°54′E	5.59	21.27	37.27	34.53	1.03	15.08	43.29	2.75	1.44	91.37	5.46	0.33	13.12	231.59
JC1	42°21′N, 126.22′E	5.59	21.08	46.48	6.45	0.74	2.61	65.68	2.05	1.48	72.73	5.70	0.31	9.37	185.76
JC2	42°21′N, 126.22′E	5.15	19.98	55.14	4.63	0.70	2.72	65.77	3.00	1.67	70.68	4.12	0.50	8.46	219.02
JC3	42°21′N, 126.22′E	5.23	18.71	60.58	4.10	0.75	2.67	60.83	3.10	1.42	123.43	3.92	0.27	9.17	166.78
JC4	42°21′N, 126.22′E	5.20	19.89	40.95	5.33	0.80	2.35	67.55	2.13	1.47	87.42	4.73	0.48	7.60	126.36
WY1	48°34′N, 129°27′E	4.95	13.84	26.14	4.63	1.39	14.94	45.53	2.68	1.46	96.37	9.76	0.51	18.56	309.35
WY2	48°34′N, 129°27′E	4.83	14.63	21.90	5.31	1.25	9.78	60.04	3.34	1.63	117.55	6.95	0.38	11.13	261.21
WY3	48°33′N, 129°28′E	4.80	9.48	39.72	6.23	0.87	5.26	64.86	4.00	1.70	99.10	5.81	1.52	9.88	319.82
TB1	48°25′N, 129°08′E	4.48	13.08	21.29	3.74	1.23	5.47	57.37	4.22	1.73	76.76	3.27	7.31	18.39	228.58
TB2	48°25′N, 129°08′E	4.84	13.53	16.32	5.04	1.23	5.29	48.82	2.44	1.45	68.87	7.96	0.10	17.21	162.62
JS	51°08′N, 124°10′E	5.21	26.83	20.48	5.60	1.94	10.90	64.32	4.62	3.33	97.76	3.43	5.11	12.90	181.27
TQ2	52°57′N, 122°52′E	5.57	22.26	18.96	4.68	1.71	15.92	68.72	3.34	1.77	79.15	6.96	6.91	17.72	965.42
HT	51°37′N, 123°59′E	4.11	12.91	27.38	5.19	0.96	7.90	73.70	3.70	1.19	50.63	2.13	1.44	4.40	61.70

*TN, total nitrogen; NH_4_^+^, ammonium; NO_3_^–^, nitrate; TP, total phosphorus; PO_4_^3–^, phosphate; OM, organic matter; DOC, dissolved organic carbon; TS, total sulfur; SO_4_^2–^, sulfate; Fe^2+^, ferrous ion; Fe^3+^, ferric ion; TFe, total iron; TMn, total manganese.*

**TABLE 2:** Physicochemical properties of water samples in peatlands (*n* = 3).

**Site**	**Latitude and longitude**	**pH**	**TN (mg L^–1^)**	**NH_4_^+^ (mg L^–1^)**	**NO_3_^–^ (mg L^–1^)**	**TP (mg L^–1^)**	**PO_4_^3–^ (mg L^–1^)**	**OM (mg L^–1^)**	**DOC (mg L^–1^)**	**SO_4_^2–^ (mg L^–1^)**	**Fe^2+^ (mg L^–1^)**	**Fe^3+^ (mg L^–1^)**	**TFe (mg L^–1^)**	**TMn (10^–3^ mg kg^–1^)**	**ORP (mV)**
QL	47°46′N, 132°54′E	7.12	2.77	1.70	0.11	0.08	0.03	126.40	13.66	10.41	0.51	0.85	1.36	3.84	120.33
HH1	47°43′N, 133°35′E	6.20	2.04	0.04	1.04	0.05	0.01	243.73	41.71	0.95	1.01	2.71	3.72	3.58	111.00
HH2	47°47′N, 133°38′E	6.20	2.98	0.02	1.02	0.15	0.05	392.33	63.08	1.30	1.18	0.46	1.63	5.48	118.00
BL	47°32′N, 133°54′E	7.08	2.44	0.05	0.97	0.13	0.06	244.37	19.14	0.93	1.02	1.16	2.51	2.27	145.67
SJZ	47°32′N, 133°54′E	6.34	2.62	0.04	1.03	0.08	0.03	279.00	59.03	0.79	1.03	1.33	2.36	4.80	124.67
JC1	42°21′N, 126°22′E	6.19	2.22	0.07	0.66	0.08	0.03	386.87	96.39	2.00	1.84	11.52	13.36	6.42	81.67
JC2	42°21′N, 126°22′E	6.17	2.37	0.07	0.86	0.07	0.02	405.30	78.87	2.03	1.91	8.58	10.49	8.71	83.00
JC3	42°21′N, 126°22′E	6.29	1.83	0.06	0.45	0.07	0.02	365.00	105.35	2.56	1.72	3.96	5.68	4.22	111.00
JC4	42°21′N, 126°22′E	6.05	2.21	0.06	0.33	0.10	0.02	400.93	34.92	2.30	2.10	7.73	9.83	12.14	93.33
WY1	48°34′N, 129°27′E	5.63	1.39	0.01	0.07	0.24	0.13	627.00	133.13	2.78	3.76	7.64	11.39	3.80	98.00
WY2	48°34′N, 129°27′E	5.17	2.18	0.03	0.18	0.26	0.12	938.13	163.20	0.98	3.84	5.29	9.13	15.63	156.33
WY3	48°33′N, 129°28′E	5.06	1.86	0.04	0.22	0.17	0.04	704.67	159.47	1.75	3.34	1.89	5.23	12.96	204.67
WY4	48°33′N, 129°28′E	5.29	1.28	0.01	0.07	0.08	0.01	510.20	61.42	1.24	1.49	1.21	2.71	16.84	106.00
TB2	48°25′N, 129°08′E	6.07	0.96	0.01	0.08	0.12	0.05	321.50	58.34	1.22	2.27	1.59	3.86	19.91	120.33
JS	51°08′N, 124°10′E	6.14	1.12	0.04	0.16	0.07	0.01	222.67	74.82	3.51	2.74	3.04	5.78	4.03	75.33
TQ1	52°57′N, 122°52′E	5.97	1.52	0.01	0.11	0.08	0.01	532.90	65.39	0.76	1.27	0.54	1.81	16.78	120.67
TQ2	52°57′N, 122°52′E	6.02	1.17	0.02	0.07	0.33	0.01	453.87	66.00	0.96	1.34	1.42	2.76	6.13	176.33
HT	51°37′N, 123°59′E	5.01	1.41	0.04	0.31	0.12	0.03	437.03	93.94	1.16	1.39	0.44	1.83	4.03	229.33

*TN, total nitrogen; NH_4_^+^, ammonium; NO_3_^–^, nitrate; TP, total phosphorus; PO_4_^3–^, phosphate; OM, organic matter; DOC, dissolved organic carbon; SO_4_^2–^, sulfate; Fe^2+^, ferrous ion; Fe^3+^, ferric ion; TFe, total iron; TMn, total manganese; T, temperature; DO, dissolved oxygen; ORP, oxidation-reduction potential.*

### Composition of Bacteria Involved in Iron Redox Cycling

**FIGURE 1:**
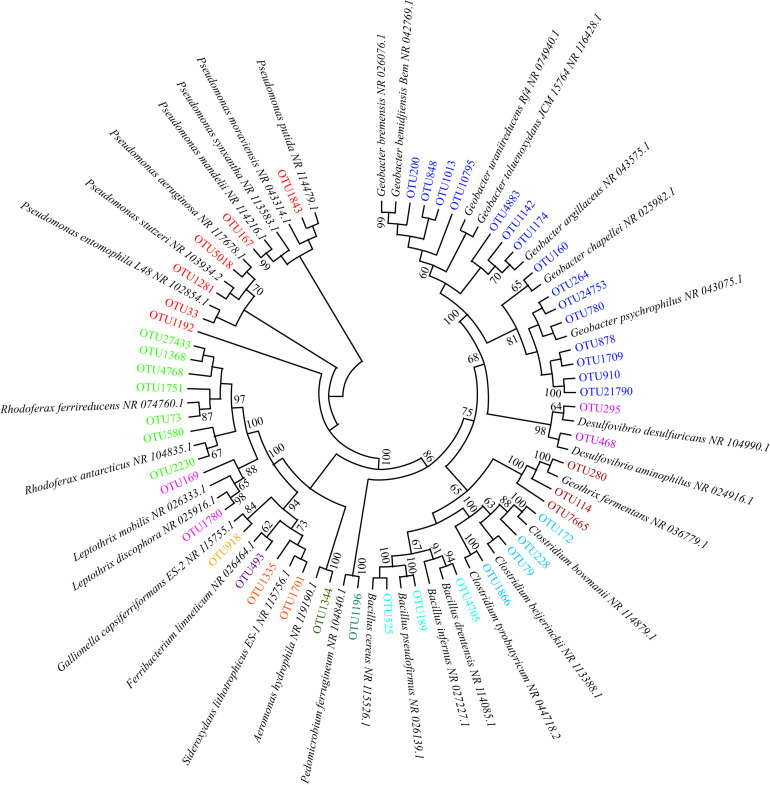
Neighbor-joining phylogenetic tree of sequences obtained from peatland soil and water. Bootstrap values are shown near the nodes (based on 1,000 replicate trees). The phylotypes (operational taxonomic units [OTUs]) with sequence similarity with known Fe(II)-oxidizing and Fe(III)-reducing bacteria greater than 97% are labeled in different colors. The phylotypes labeled in the same color represent the same genus. For example, the phylotypes labeled in red represent *Pseudomonas*.

**FIGURE 2:**
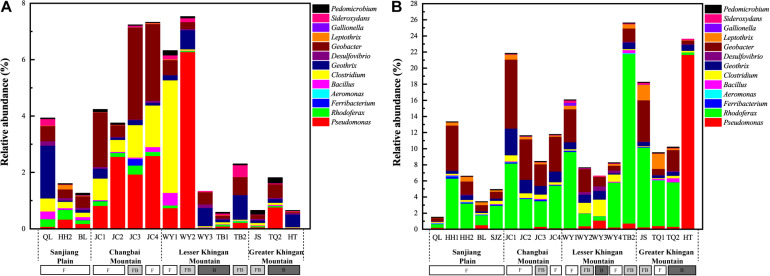
The relative abundance of iron redox cycling bacteria in soil **(A)** and water **(B)**. F, fen; FB, fen–bog complex; B, bog.

We identified 48 phylotypes showing >97% sequence similarity with verified FeOB and FeRB across the NCBI database. These OTUs belonged to four genera of FeOB and nine genera of FeRB (Figure 1). *Sideroxydans* and *Pedomicrobium* were the most enriched genera among the FeOB, while *Geobacter*, *Geothrix*, *Clostridium*, *Rhodoferax*, and *Pseudomonas* were the most enriched genera among the FeRB. The relative abundance of FeRB was approximately one to two orders of magnitude higher than that of FeOB at all sites. On average, FeOB accounted for 0.15% and 0.56% of the total bacteria in soil and water, respectively, and FeRB for 3.15% and 11.05%, respectively (Figure 2).

**FIGURE 3:**
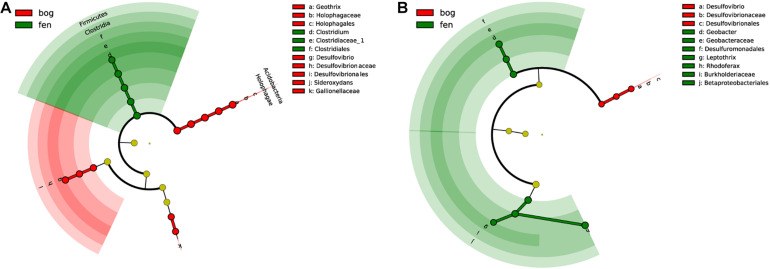
Cladograms indicating the phylogenetic distribution of iron redox cycling bacterial lineages associated with regions in soil **(A)** and water **(B)**. Lineages were identified by linear discriminant analysis effect size (LEfSe) using a LDA score threshold of over 4.0. Circles represent phylogenetic levels from domain to species inside out. Labels are shown for the phylum and genus levels.

Cladogram analysis revealed that several groups of iron redox cycling bacteria in soil and water were associated with peatland type (Figure 3). In soil samples, the relative abundance of *Clostridium* was greatly enriched in fens. The relative abundances of *Geothrix*, *Desulfovibrio*, and *Sideroxydans* were markedly enriched in bogs (Figure 3A). In water samples, the relative abundances of *Rhodoferax*, *Geobacter*, and *Leptothrix* were highest in fen peatland, while that of *Desulfovibrio* was highest in bog (Figure 3B).

### Assembly of Iron Redox Cycling Bacteria

**FIGURE 4:**
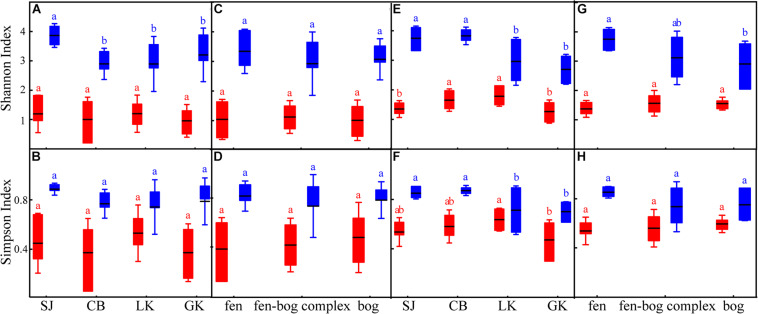
The alpha diversity of iron redox cycling bacteria in soil **(A–D)** and water **(E–H)** of the different regions (Sanjiang Plain [SJ], Changbai Mountain [CB], Lesser Khingan Mountain [LK], and Greater Khingan Mountain [GK]) and peatland types (fen, fen–bog complex, bog). The red box represents iron-oxidizing bacteria; the blue box represents iron-reducing bacteria. Different letters indicate statistical significance among the different levels.

Overall, we observed that community diversity was higher in FeRB than in FeOB in both water and soil samples (Figure 4). In soil samples, FeRB diversity was greatest in SJ. In water samples, FeRB diversity was significantly greater in SJ than in GK (*p* < 0.05). Meanwhile, the Shannon indices of fen FeRB were significantly greater than those of bog FeRB (*p* < 0.05). In addition, FeRB diversity in soil samples was greatest in fen. The above results indicated that the community diversity of iron redox cycling bacteria was correlated with distribution and peatland type.

**FIGURE 5:**
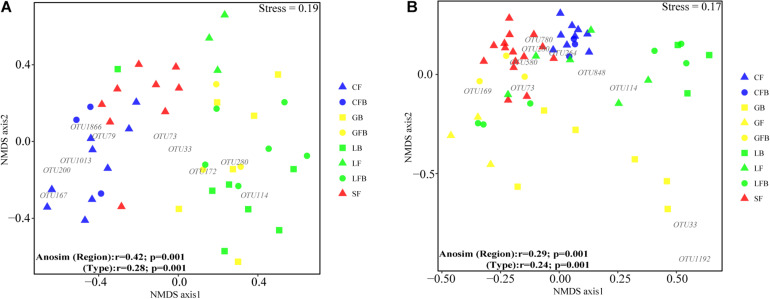
Non-metric multidimensional scaling (NMDS) and two-way analysis of similarity (ANOSIM) of bacterial communities involved in iron redox cycling in soil **(A)** and water **(B)** based on Bray–Curtis dissimilarity. Red, blue, green, and yellow represent Sanjiang Plain, Changbai Mountain, Lesser Khingan Mountain, and Great Khingan Mountain, respectively. Triangles, circles, and squares represent fen, fen–bog complex, and bog, respectively. For example, blue triangles represent the fen in Changbai Mountain.

Water-derived iron redox cycling bacterial communities showed no differences between region and peatland type (Figure 5B). Interestingly, regional distribution (*r* = 0.42, *p* = 0.001) had a greater effect on soil-derived iron redox cycling bacterial communities than peatland type (*r* = 0.28, *p* = 0.001) (Figure 5A).

We found that the similarity among observed communities was largely distinguishable from that of the null expectation for water, such as the water samples obtained from LK (*p* = 0.002) and Greater Khingan Mountain (*p* = 0.003). However, no notable differences (*p* > 0.05) were detected between the similarity among observed communities and that of the null expectation for soil. In addition, the similarity among observed communities was distinguishable from that of the null expectation for all peatland types (*p* < 0.05), except for soil samples from fen–bog complex and bog. The SES value was greater than zero in the samples from both soil and water, indicating deterministic and stochastic processes involved in the assembly of iron redox cycling bacteria, although their relative importance differed (Table 3).

**TABLE 3:** Significance test of the similarity between the observed bacterial communities and null model simulations and the standardized effect size (SES) across different sampling regions and peatland types based on weighted Bray–Curtis distances for iron redox cycling bacterial communities.

	**Region and type**	**Mean of observed similarity**	**Mean of null expected similarity**	***F***	***p***	**SES**	**Ecological processes**
**Soil**	Sanjiang Plain	0.430	0.545	2.862	0.117	1.724	Stochasticity
	Changbai Mountain	0.400	0.458	0.889	0.341	0.694	Stochasticity
	Lesser Khingan Mountain	0.277	0.351	1.220	0.256	1.865	Stochasticity
	Great Khingan Mountain	0.362	0.363	0.004	0.943	0.087	Stochasticity
	Fen	0.297	0.448	12.310	**0.001**	2.533	Determinism
	Fen–bog complex	0.217	0.302	3.075	0.086	2.036	Stochasticity
	Bog	0.333	0.398	1.096	0.301	0.725	Stochasticity
**Water**	Sanjiang Plain	0.449	0.523	1.942	0.189	2.277	Stochasticity
	Changbai Mountain	0.628	0.651	0.132	0.705	0.273	Stochasticity
	Lesser Khingan Mountain	0.410	0.587	14.249	**0.002**	2.513	Determinism
	Great Khingan Mountain	0.370	0.594	10.443	**0.003**	2.318	Determinism
	Fen	0.426	0.531	5.785	**0.029**	2.027	Determinism
	Fen–bog complex	0.461	0.613	9.395	**0.004**	2.288	Determinism
	Bog	0.299	0.533	14.983	**0.001**	2.909	Determinism

*Permutational multivariate analysis of variance (PERMANOVA) was conducted. *p*-values <0.05 were in bold.*

### Distribution Pattern of Iron Redox Cycling Bacteria

**FIGURE 6:**
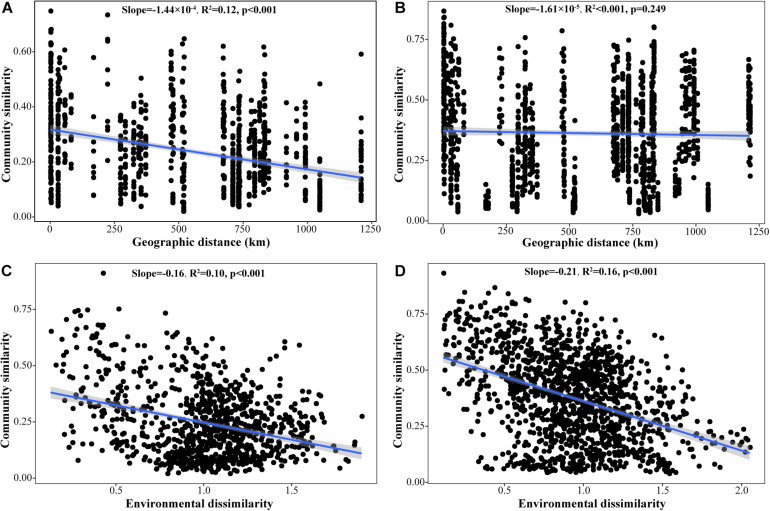
Distance–decay relationships among iron redox cycling bacterial community similarity, geographic distance, and environmental dissimilarity for all sites in soil **(A,C)** and water **(B,D)**. Community similarity was calculated by 1-Bray–Curtis dissimilarity. Environmental dissimilarity was calculated by Euclidean distance.

The pairwise similarity among iron redox cycling bacterial communities in soil samples decreased significantly with increasing geographic distance and environmental heterogeneity, demonstrating that their variations were attributable to the joint effects of dispersal limitation and environmental selection. However, iron redox cycling bacterial community similarity in water samples was not correlated with geographic distance (*p* = 0.249) (Figure 6). In addition, Mantel path analysis showed that, in soil, the correlation coefficients of regions and peatland types with FeOB/FeRB community composition were greater than those between environmental factors and FeOB/FeRB community composition (Supplementary Figure 1A). However, the opposite trend was observed in water samples (Supplementary Figure 1B), indicating that the FeOB/FeRB community composition in water was more directly influenced by environmental factors.

**FIGURE 7:**
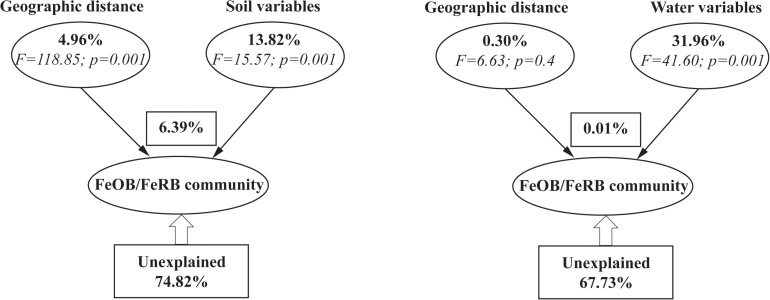
Variation partitioning analysis of the effects of geographic distance and environmental variables on the phylogenetic structure of iron redox cycling bacterial communities.

**FIGURE 8:**
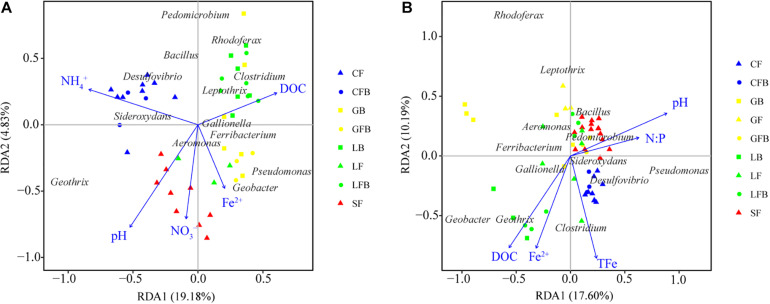
Redundancy analysis of bacterial communities involved in iron redox cycling and environmental variables in soil **(A)** and water **(B)**. Red, blue, green, and yellow represent Sanjiang Plain, Changbai Mountain, Lesser Khingan Mountain, and Great Khingan Mountain, respectively. Triangles, circles, and squares represent fen, fen–bog complex, and bog, respectively. For example, the blue triangle represents the fen in Changbai Mountain.

Variance partitioning analysis (VPA) was used to quantify the relative contributions of environmental factors and spatial limitation to the community structure of iron redox cycling bacteria (Figure 7). Physicochemical properties and geographic distance explained 25.18% and 33.37% of the observed variation in soil and water, respectively. The contribution of geographic distance to the iron redox cycling bacterial community was significantly higher in soil (4.96%) than in water (0.30%). Regardless of whether samples were collected in soil or water, the relative contributions of physicochemical properties were significantly higher than those of geographic distance. RDA showed that pH appeared to be an important factor regulating the composition of iron redox cycling bacterial communities in both soil and water (Figure 8). For nutrients, NH_4_^+^, DOC, Fe^2+^, and NO_3_^–^ contributed more to mediate iron redox cycling bacterial communities in soil, while water-related bacterial communities involved in iron redox cycling were mainly affected by N: P, DOC, TFe, and Fe^2+^.

## Discussion

### The Response of Iron Redox Cycling Bacteria to Physicochemical Properties

Several studies have reported that microbes are the primary oxidizers in anaerobic environments or environments with an oxygen content of less than 50 μM (Rentz et al., 2007; Chan et al., 2016). Additionally, we previously found that the relative abundance of iron redox cycling bacteria increased with increased soil moisture content during wetland restoration (Yang et al., 2019a). In the current study, we observed that the relative abundances of FeOB and FeRB were higher in flooded environments than in water-saturated environments. Intriguingly, minerotrophic fens were mostly flooded and ombrotrophic bogs were mostly water-saturated in our sampling sites. We speculated that the distribution of iron redox cycling bacteria may have been related to the type of peatland, which was consistent with the changing patterns of relative abundance and diversity of the iron redox cycling bacteria (Figures 4, 5). For instance, the relative abundances of FeOB and FeRB in the peatlands of LK were both ranked in the order fen > fen–bog complex > bog (Figure 2). These findings demonstrated that iron redox cycling bacteria were more abundant in reducing conditions and may reflect the alternation of dry and wet conditions and hydrological fluctuation in peatlands (Küsel et al., 2008). The Fe cycle can be accompanied by migration and transformation of C, N, and P, indicating that iron redox cycling bacteria are relevant to ecological stoichiometry (Melton et al., 2014; Downie et al., 2018). Phosphorus has a strong affinity for iron oxyhydroxides, especially those associated with the sheaths of FeOB (Rentz et al., 2009). In addition, most nitrate-reducing FeOB require an organic co-substrate, such as acetate, to continually oxidize Fe(II) to Fe(III) (Muehe et al., 2009). In this study, the NMDS analysis revealed that the C:N and N:P ratios were significantly correlated with iron redox cycling bacteria (Supplementary Table 2). Du et al. (2020) evaluated the spatial characteristics of global N and P limitation based on the ratio of site-averaged leaf N and P resorption efficiencies of the dominant species and revealed that the natural terrestrial land area in Northeast China could be co-limited by N and P or weakly limited by either nutrient alone. This suggested that the interaction and balance of nutrient restricted individuals and communities of iron redox cycling bacteria. Furthermore, the results of the RDA revealed that NH_4_^+^ was also an important factor for the iron redox cycling bacterial community. Ammonium concentration is closely associated with iron redox cycling bacteria, both as an electron donor for iron oxidation and as a product of metabolism for use in iron reduction. For instance, *Geothrix* has been suggested to couple FeS_2_ oxidation to NO_3_^–^ reduction (Haaijer et al., 2007). Moreover, Zhou et al. (2016) recently reported that FeRB such as *Geobacter* play key roles in the Feammox (anaerobic ammonium oxidation coupled to iron(III) reduction) process.

We also observed that DOC and Fe^2+^ concentrations were common factors shaping the structure of iron redox cycling bacterial communities in both soil and water samples (Figure 8). DOC can not only constitute a carbon source for the growth of microorganisms but also improve the accessibility of other elements for microorganisms (Marschner and Kalbitz, 2003; Stenson, 2009). Daugherty et al. (2017) showed that complexation by organic ligands can increase the bioavailability of Fe(III). Besides, DOC can also be used as an electron donor and acceptor for repeated iron redox cycling (Klüpfel et al., 2014; Kügler et al., 2019). Intriguingly, the effect of pH on the regulation of community structure observed in our study was not in line with previously reported results. Numerous studies have documented that pH range is the primary factor regulating bacterial community composition. However, we observed that, although pH was indeed one of the influencing factors, it was not the most important limiting factor in shifting redox-cycling bacterial communities, even with variations in pH ranges of 1.48 and 2.11 units in soil and water samples, respectively. These results indicated that the selective pressure of environmental factors on whole bacterial communities or functional microorganisms such as iron redox cycling bacterial communities might be variable (Liu et al., 2016; Shi et al., 2020).

### Indicative Taxa Across the Peatlands of Northeast China

Based on recently reported definitions of rare taxa (Xue et al., 2018), we found that 72.9%–87.5% of OTUs associated with iron redox cycling bacterial belonged to rare taxa (Supplementary Table 3). Zhou et al. (2020) demonstrated that rare bacterial species are more sensitive than abundant taxa to environmental changes. Therefore, iron redox cycling bacteria might play indicative roles in peatlands. In this study, we identified four genera of indicator bacteria in soil and four in water among the different peatland types (Figure 3).

Hydrological conditions restrict the distribution of some microbial groups (Yang et al., 2019b). Although the soil samples were collected on the surface layer in this study, fen soil samples presented relatively reducing conditions due to the flooded conditions. In contrast, soil samples from bog exposed to the air exhibited relatively oxidizing conditions. As obligate anaerobic microorganisms, *Clostridium* and *Geobacter* are better suited to anoxic environments such as those found in wetlands, freshwater sediments, and oceans (Mohr et al., 2013; Zhou et al., 2014), likely explaining why the relative abundances of *Clostridium* in soil and *Geobacter* in water were higher in fens than in bogs. Finneran et al. (2003) found that *Rhodoferax* are facultative anaerobic bacteria that can oxidize acetate *via* the reduction of Fe(III), which might explain why *Rhodoferax* was enriched in fen water samples.

Except for *Desulfovibrio*, we found that the other indicator species in the water and soil showed inconsistencies. Based on the database of Functional Annotation of Prokaryotic Taxa (FAPROTAX) created by Louca et al. (2016), these indicator species are connected to the C cycle (ligninolysis, aromatic compound degradation, and fumarate respiration), N cycle (nitrification, denitrification, and nitrite ammonification), and S cycle (sulfate respiration, dissimilatory reduction of elemental sulfur, sulfite oxidation, and respiration). Our study showed that C concentration was significantly correlated with *Geothrix* and *Sideroxydans*, while N and S concentrations were significantly correlated with *Rhodoferax*, *Desulfovibrio*, *Geothrix*, and *Sideroxydans*. Intriguingly, we found that the genera *Geobacter*, *Desulfovibrio*, *Geothrix*, and *Sideroxydans* were significantly related to Fe^2+^ or total Fe (Supplementary Table 4). These results imply that metabolism may vary according to the microbial group, and further study is needed to identify potential interactions between those indicative taxa and link them to community composition and ecological functions.

### Deterministic and Stochastic Processes in the Structuring of Iron Redox Cycling Bacterial Communities

The ecological mechanisms (determinism vs. stochasticity) associated with community assembly play important roles in shaping microbial community composition and structure. In general, a deterministic process is a non-random and niche-based ecological process, including environmental filtering and various biological interactions (e.g., competition, facilitation, mutualisms, and predation). However, a stochastic process is a random ecological process, including probabilistic dispersal, random speciation and extinction, and ecological drift (Stegen et al., 2012; Zhou and Ning, 2017). In our study, SES statistics confirmed that both deterministic and stochastic processes were involved in the assembly of bacterial communities involved in iron redox cycling. However, the DDR analysis indicated that deterministic processes played a more important role in regulating iron redox cycling-related bacterial communities in water compared with that in soil. Intriguingly, null model tests revealed that deterministic processes were dominant in water samples across the different peatland types, whereas stochastic processes substituted deterministic processes from minerotrophic fens to ombrotrophic bogs in soils. This indicated that iron redox cycling bacteria in soil were more likely to be governed by dispersal limitation. The differences in assembly mechanisms between iron redox cycling bacteria in water and soil across peatlands were mainly due to the different microbial types present as a result of environmental conditions (Wang et al., 2017; Mo et al., 2018). Zhou et al., 2002) proved that water disrupted the spatial isolation of soil bacteria and increased the interaction of bacterial communities; thus, we speculate that the dispersal limitation of iron redox cycling bacteria in soil could be alleviated by water.

Notably, iron redox cycling bacteria in soil and water were affected by environmental factors and spatial patterns, with the former having a greater impact than the latter (Figure 7). This was consistent with previous results reported in the black soils of northeast China (Liu et al., 2014, 2016). Nevertheless, the contribution of environmental variables was greater in water (31.96%) than in soil (13.82%), and the contribution of region was substantially greater in soil (4.96%) than in water (0.30%). One explanation may be that the soil environment is more complicated due to larger variations in temperature, hydrology, and nutrition, among other factors (Nie et al., 2009; Peralta et al., 2014; Wu et al., 2015). In contrast, the fluidity and solubility of water may, to some extent, homogenize environmental conditions (Jäger et al., 2016). Mantel path analysis also indicated that water-derived bacterial communities associated with iron redox cycling were more affected by environmental factors than by region or type of peatland, while the soil samples showed the opposite trend (Supplementary Figure 1). Consistent with this, DDR analysis indicated that iron redox cycling bacterial communities were mainly affected by environmental factors and that environmental factors exerted a more direct effect on iron redox cycling bacterial communities in water. These results indicated that environmental factors were more important than geographic location in determining the distribution of iron redox cycling bacterial communities in peatlands.

## Conclusion

This study was the first to investigate the biogeographic distribution and mechanism of assembly of bacterial communities associated with iron redox cycling in surface soil and water samples collected from peatlands. Iron redox cycling bacteria were detected in all the samples; however, iron-reducing bacteria showed higher relative abundance and diversity than FeOB. To some extent, *Rhodoferax*, *Clostridium*, *Geothrix*, *Sideroxydans*, *Geobacter*, *Desulfovibrio*, and *Leptothrix* could be used as bioindicators in peatlands for characterizing different hydrological conditions and nutrient demands. Overall, DOC, Fe^2+^, and pH were the key factors affecting the abundance and community composition of iron redox cycling bacteria. Distance–decay analysis indicated that bacterial communities associated with iron redox cycling in water displayed greater environmental heterogeneity, while those in soil displayed greater spatial limitation. Null model analysis based on species distribution further revealed that stochastic and deterministic processes played important roles in mediating the assembly of bacterial communities involved in iron redox cycling in soil and water, respectively.

## Data Availability Statement

The data analyzed in this study is subject to the following licenses/restrictions: Further inquiries can be directed to the corresponding author. Requests to access these datasets should be directed to LY, yangliang@iga.ac.cn.

## Author Contributions

LY contributed to the investigation, methodology, and writing—original draft. MJ contributed to the supervision, funding acquisition, conceptualization, and writing—review and editing. YZ contributed to the funding acquisition and validation. LQ and YC contributed to the sampling and formal analysis. All authors contributed to the article and approved the submitted version.

## Conflict of Interest

The authors declare that the research was conducted in the absence of any commercial or financial relationships that could be construed as a potential conflict of interest.

## Footnotes

1 https://ccb.jhu.edu/software/FLASH/

2 http://www.arb-silva.de

3 http://www.ncbi.nlm.nih.gov

4 http://www.mothur.org

## Supplementary Material

Supplementary Figure 1 Mantel path analysis linking the taxonomic composition of bacterial communities involved in iron redox cycling to different regions, peatland types, and physicochemical properties. **(A)** Soil; **(B)** water. Solid lines represent Mantel correlation coefficients; *p*-values are in parentheses.
