# Supplementary figures and images for: Geographical Distribution of Iron Redox Cycling Bacterial Community in Peatlands: Distinct Assemble Mechanism Across Environmental Gradient
Source: Front Microbiol. 2021 May 25;12:674411. doi: 10.3389/fmicb.2021.674411 (PMC8185058; doi:10.3389/fmicb.2021.674411)

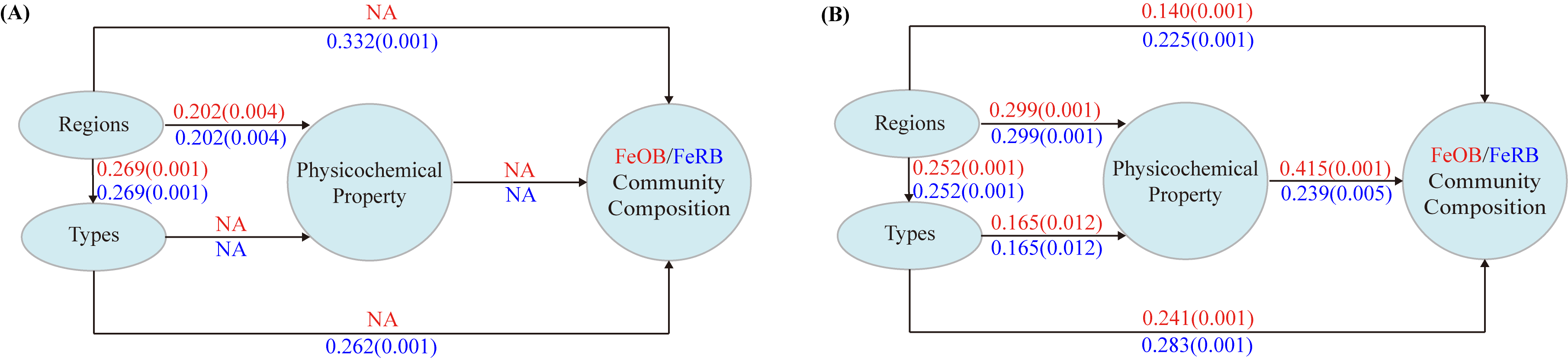

Supplement: Supplementary Figure 1 — Mantel path analysis linking the taxonomic composition of bacterial communities involved in iron redox cycling to different regions, peatland types, and physicochemical properties. (A) Soil; (B) water. Solid lines represent Mantel correlation coefficients; p-values are in parentheses. [file Image_1.TIF]
