# Supplementary material for: Geographical Distribution of Iron Redox Cycling Bacterial Community in Peatlands: Distinct Assemble Mechanism Across Environmental Gradient
Source: Front Microbiol. 2021 May 25;12:674411. doi: 10.3389/fmicb.2021.674411 (PMC8185058; doi:10.3389/fmicb.2021.674411)
Supplement: Supplementary file 2 [file Table_1.doc]

**TABLE S1 | Details of sampling sites in peatlands.**

| Region | Site | Location | Latitude & longtitude | Peat type | Water condition | Dominant vegetation | Soil sampling | Water sampling |
| --- | --- | --- | --- | --- | --- | --- | --- | --- |
| Sanjiang Plain | QL | Qinglong, Heilongjiang | 47°46′N, 132°54′E | fen | Waterlogged 10-20 cm | *Carex spp., Deyeuxia angustifolia* | 0-30cm | Pore water 0-30cm |
|  | HH1 | Honghe 1, Heilongjiang | 47°43′N, 133°35′E | fen | Waterlogged 40-45 cm | *Carex spp., Deyeuxia angustifolia* | None | Surface water 40-45cm |
|  | HH2 | Honghe 2, Heilongjiang | 47°47′N, 133°38′E | fen | Waterlogged 10-20 cm | *Carex spp., Deyeuxia angustifolia* | 0-30cm | Surface water 10-20 cm |
|  | BL | Bilahong, Heilongjiang | 47°32′N, 133°54′E | fen | Waterlogged 10-20 cm | *Carex spp., Deyeuxia angustifolia* | 0-30cm | Pore water 0-30cm |
|  | SJZ | Jiansanjiang, Heilongjiang | 47°32′N, 133°54′E | fen | Waterlogged 40-45 cm | *Carex spp., Deyeuxia angustifolia* | None | Surface water 40-45 cm |
| Changbai Mountain | JC1 | Jichuan 1, Jilin | 42°21′N, 126°22′E | fen | Waterlogged 15-20 cm | *Carex spp., Betula fruticosa* | 0-30cm | Surface water 15-20 cm |
|  | JC2 | Jichuan 2, Jilin | 42°21′N, 126°22′E | fen | Waterlogged 15-20 cm | *Carex spp., Phragmites australis* | 0-30cm | Surface water 15-20 cm |
|  | JC3 | Jichuan 3, Jilin | 42°21′N, 126°22′E | fen-bog complex | Waterlogged 5-10 cm | *Carex spp.,* *Sphagnum* | 0-30cm | Surface water 5-10 cm |
|  | JC4 | Jichuan 4, Jilin | 42°21′N, 126°22′E | fen | Waterlogged 15-20 cm | *Carex spp., Deyeuxia angustifolia* | 0-30cm | Surface water 15-20 cm |
| Lesser Khingan Mountain | WY1 | Wuyi 1, Heilongjiang | 48°34′N, 129°27′E | fen | Waterlogged 20-25 cm | *Carex spp.,* *Alnus mandshurica* | 0-30cm | Surface water 20-25 cm |
|  | WY2 | Wuyi 2, Heilongjiang | 48°34′N, 129°27′E | fen-bog complex | Waterlogged 5-10 cm | *Carex spp., Sphagnum*, *Alnus mandshurica* | 0-30cm | Surface water 5-10 cm |
|  | WY3 | Wuyi 3, Heilongjiang | 48°33′N, 129°28′E | bog | Saturated | *Sphagnum, Ledum palustre L., Vaccinium uliginosum Linn.* | 0-30cm | Pore water 0-30cm |
|  | WY4 | Wuyi 4, Heilongjiang | 48°33′N, 129°28′E | fen | Waterlogged 50-70 cm | *Carex spp., Deyeuxia angustifolia, Betula fruticosa* | None | Surface water 50-70 cm |
|  | TB1 | Tangwanghe 1, Heilongjiang | 48°25′N, 129°08′E | bog | Saturated | *Sphagnum, Ledum palustre L., Vaccinium uliginosum Linn.* | 0-30cm | None |
|  | TB2 | Tangwanghe 2, Heilongjiang | 48°25′N, 129°08′E | fen-bog complex | Waterlogged 15-20 cm | *Carex spp., Sphagnum*, *Betula fruticosa* | 0-30cm | Surface water 15-20 cm |
| Greater Khingan Mountain | JS | Jinsong, Heilongjiang | 51°08′N, 124°10′E | fen-bog complex | Saturated | *Carex spp., Deyeuxia angustifolia* | 0-30cm | Pore water 0-30cm |
|  | TQ1 | Tuqiang 1, Heilongjiang | 52°57′N, 122°52′E | fen | Waterlogged 50-60 cm | *Carex spp.,* *S. Rosmarinifolia L. Var brachypoda* | None | Surface water 50-60 cm |
|  | TQ2 | Tuqiang 2, Heilongjiang | 52°57′N, 122°52′E | bog | Saturated | *Sphagnum, Ledum palustre L., Vaccinium uliginosum Linn.* | 0-30cm | Pore water 0-30cm |
|  | HT | Hongtu, Heilongjiang | 51°37′N, 123°59′E | bog | Saturated | *Sphagnum, Ledum palustre L., Vaccinium uliginosum Linn.* | 0-30cm | Pore water 0-30cm |
