# Supplementary material for: Geographical Distribution of Iron Redox Cycling Bacterial Community in Peatlands: Distinct Assemble Mechanism Across Environmental Gradient
Source: Front Microbiol. 2021 May 25;12:674411. doi: 10.3389/fmicb.2021.674411 (PMC8185058; doi:10.3389/fmicb.2021.674411)
Supplement: Supplementary file 3 [file Table_2.doc]

**TABLE S2 | Pairwise regression between NMDS1 scores and NMDS2 scores and soil factors.**

|  |  | **pH** | **NH_4_^+^** | **DOC** | **Fe^2+^** | **Fe^3+^** | **TFe** | **C:N** | **N:P** |
| --- | --- | --- | --- | --- | --- | --- | --- | --- | --- |
| Soil | NMDS1 | -0.437** | -0.621** | 0.521** | NA | 0.390** | 0.327* | 0.424** | -0.768** |
|  | NMDS2 | 0.312* | NA | NA | 0.342* | NA | 0.334* | -0.370* | NA |
| Water | NMDS1 | -0.695** | NA | 0.624** | 0.507** | NA | 0.300* | 0.453** | -0.348** |
|  | NMDS2 | -0.299* | -0.447** | NA | NA | -0.539** | -0.520** | 0.408** | -0.436** |

Significant test were performed: *p<0.05; ** p<0.01.
