# Supplementary material for: Geographical Distribution of Iron Redox Cycling Bacterial Community in Peatlands: Distinct Assemble Mechanism Across Environmental Gradient
Source: Front Microbiol. 2021 May 25;12:674411. doi: 10.3389/fmicb.2021.674411 (PMC8185058; doi:10.3389/fmicb.2021.674411)
Supplement: Supplementary file 4 [file Table_3.doc]

**TABLE S3 | Rare taxa in soil and water of peatlands.**

| **Taxon** | **soil** | **water** |  | **Taxon** | **soil** | **water** |  | **Taxon** | **soil** | **water** |
| --- | --- | --- | --- | --- | --- | --- | --- | --- | --- | --- |
| OTU1843 | RT | CART |  | OTU189 | RT | RT |  | OTU1709 | RT | RT |
| OTU167 | CART | CART |  | OTU4705 | RT | RT |  | OTU200 | CART | RT |
| OTU5018 | RT | RT |  | OTU1866 | CART | RT |  | OTU21790 | RT | RT |
| OTU1281 | RT | RT |  | OTU79 | CART | RT |  | OTU24753 | RT | RT |
| OTU33 | CART | CART |  | OTU228 | RT | RT |  | OTU264 | RT | CART |
| OTU1192 | RT | CART |  | OTU172 | RT | RT |  | OTU4883 | RT | RT |
| OTU27433 | RT | RT |  | OTU7665 | CART | RT |  | OTU780 | RT | CART |
| OTU1368 | RT | CART |  | OTU114 | RT | CART |  | OTU848 | RT | CART |
| OTU4768 | RT | RT |  | OTU280 | RT | CART |  | OTU878 | RT | RT |
| OTU1751 | RT | RT |  | OTU468 | RT | RT |  | OTU910 | RT | RT |
| OTU73 | RT | CART |  | OTU295 | RT | RT |  | OTU169 | RT | CART |
| OTU580 | RT | RT |  | OTU1013 | RT | RT |  | OTU1780 | RT | RT |
| OTU2230 | RT | RT |  | OTU10795 | RT | RT |  | OTU918 | RT | RT |
| OTU493 | RT | RT |  | OTU1142 | RT | RT |  | OTU1335 | RT | RT |
| OTU1344 | RT | RT |  | OTU1174 | RT | RT |  | OTU1701 | RT | RT |
| OTU525 | RT | RT |  | OTU160 | RT | CART |  | OTU1196 | RT | RT |

Rare taxa (RT) were defined as the OTUs with a relative abundance <0.01% in some samples but never abundant (≥ 1%) in any sample. Conditionally abundant and rare taxa (CART) were defined as the OTUs with a relative abundance varying from rare (< 0.01%) to abundant (≥ 1%).
