# Supplementary material for: Geographical Distribution of Iron Redox Cycling Bacterial Community in Peatlands: Distinct Assemble Mechanism Across Environmental Gradient
Source: Front Microbiol. 2021 May 25;12:674411. doi: 10.3389/fmicb.2021.674411 (PMC8185058; doi:10.3389/fmicb.2021.674411)
Supplement: Supplementary file 5 [file Table_4.doc]

**TABLE S4 | Pearson correlations between relative abundances of iron redox cycling bacterial genus and environmental factors.**

|  |  | pH | TN | NH_4_^+^ | NO_3_^-^ | TP | PO_4_^3-^ | TOC | DOC | Fe^2+^ | Fe^3+^ | TFe | SO_4_^2-^ | Mn | C:N | N:P |
| --- | --- | --- | --- | --- | --- | --- | --- | --- | --- | --- | --- | --- | --- | --- | --- | --- |
| *Rhodoferax* | soil | 0.459** | 0.360* | 0.359* | NA | NA | NA | NA | -0.427** | NA | -0.387** | NA | 0.317* | NA | -0.423** | 0.438** |
|  | water | NA | -0.462** | NA | NA | NA | NA | NA | NA | NA | NA | NA | -0.276* | 0.282* | NA | NA |
| *Clostridium* | soil | NA | NA | NA | NA | NA | NA | NA | NA | NA | NA | NA | NA | NA | NA | NA |
|  | water | -0.510** | NA | NA | NA | NA | NA | 0.572** | 0.627** | 0.443** | NA | NA | NA | 0.378** | 0.366** | NA |
| *Geothrix* | soil | NA | -0.315* | NA | .369* | 0.310* | 0.342* | -0.405** | NA | 0.442** | NA | 0.351* | 0.605** | NA | NA | -0.380* |
|  | water | NA | NA | NA | NA | NA | NA | NA | 0.285* | NA | 0.680** | 0.641** | NA | NA | NA | NA |
| *Geobacter* | soil | NA | NA | .524** | NA | -0.362* | -0.315* | NA | -0.300* | NA | NA | NA | NA | NA | NA | 0.416** |
|  | water | NA | NA | NA | NA | NA | NA | NA | NA | NA | 0.661** | 0.620** | NA | NA | NA | NA |
| *Leptothrix* | soil | NA | 0.448** | NA | NA | NA | NA | NA | -0.320* | NA | NA | NA | NA | NA | NA | 0.333* |
|  | water | NA | NA | NA | NA | NA | NA | NA | NA | NA | NA | NA | NA | NA | NA | NA |
| *Sideroxydans* | soil | NA | NA | -0.357* | NA | 0.325* | NA | -0.441** | NA | 0.505** | NA | 0.468** | 0.321* | NA | NA | -0.398** |
|  | water | NA | -0.381** | -0.304* | NA | NA | NA | NA | NA | 0.371** | NA | NA | NA | NA | 0.274* | -0.330* |
| *Desulfovibrio* | soil | NA | NA | NA | 0.473** | NA | NA | NA | NA | NA | NA | NA | 0.431** | NA | NA | NA |
|  | water | -0.581** | NA | -0.311* | NA | NA | NA | 0.585** | 0.503** | 0.425** | NA | NA | NA | 0.447** | 0.514** | -0.393** |

Significant test were performed: *p<0.05; ** p<0.01.
